# Ruxolitinib and vedolizumab salvage therapy in the setting of cytomegalovirus colitis for a patient with newly diagnosed very early onset inflammatory bowel disease

**DOI:** 10.1002/jpr3.70106

**Published:** 2025-10-30

**Authors:** Alison Laxer, Morris Edelman, Julie Gallagher, Benjamin Sahn

**Affiliations:** ^1^ Division of Pediatric Gastroenterology Cohen Children's Medical Center New Hyde Park New York USA; ^2^ Division of Pediatric Pathology Cohen Children's Medical Center New Hyde Park New York USA

**Keywords:** acute severe colitis, combination therapy, primary nonresponse to antitumor necrosis factor‐α

## Abstract

Acute severe ulcerative colitis increases the risk for cytomegalovirus (CMV) infection, particularly with the use of immunocompromising medications. We report a case of a 4‐year‐old with newly diagnosed very early onset inflammatory bowel disease presenting with acute severe colitis refractory to both corticosteroids and infliximab, whose course was complicated by CMV colitis. Clinical remission was achieved with combination salvage therapy of ruxolitinib and vedolizumab. Clearance of CMV was not impaired with this regimen. Initiating combination Janus Kinase inhibition and anti‐integrin salvage therapy in the setting of CMV infection and acute severe colitis is not previously described.

## 1 INTRODUCTION

Patients with acute severe colitis (ASC), particularly those treated with immunocompromising medications, are at an increased risk of disease complicated by cytomegalovirus (CMV) infection, which may result in further colonic damage.^1^ This is treated with antiviral medication, along with the reduction of intensity of immunocompromising medications.^1^, ^2^ CMV is a ubiquitous herpes virus that can result in primary infection and exhibits properties of latency and reactivation. When CMV colitis is identified in the ASC setting, its significance in worsening the underlying colitis is unclear. Initiating combination Janus Kinase inhibition and anti‐integrin salvage therapy in the setting of CMV infection and ASC is not previously described.

## 2 CASE REPORT

A 4‐year‐old 16 kg female presented in January 2024, 10 days after the onset of acute abdominal pain, bloody diarrhea, and urgency. At presentation, the pediatric ulcerative colitis activity index (PUCAI) score was 85. Infectious stool testing was negative, and laboratory tests were remarkable for anemia, hypoalbuminemia, elevated serum inflammatory markers, and fecal calprotectin of 1140 µg/g. Magnetic Resonance Enterography did not demonstrate small bowel disease. Upper endoscopy was unremarkable, and colonoscopy revealed severe pancolitis with a normal terminal ileum and histopathology consistent with chronic active pancolitis without histologic findings of granuloma. As part of a multidisciplinary approach, immunology was consulted, and an immunologic evaluation, including testing for chronic granulomatous disease, was not consistent with a quantitative or functional immune defect. Genetic evaluation consisted of a targeted panel for a primary immunodeficiency including known causative monogenic defects for very early onset inflammatory bowel disease (VEO‐IBD), which did not reveal any pathogenic mutations or variants of unknown significance consistent with the patient's phenotype. A diagnosis of VEO‐IBD with ASC was made.

Intravenous corticosteroids, 4 mg every 8 h of methylprednisolone (1 mg/kg/day of prednisone equivalent), were instituted without symptomatic response. After 3 days, infliximab 200 mg (13 mg/kg) was administered with initial improvement in PUCAI to 30 on induction Day 2. Symptoms returned by induction Day 4 and she received a second dose of infliximab on induction Day 7. An infliximab trough was 25 µg/mL at the time of the second infusion, which was followed by 2 days of clinical improvement, however she then developed frequent bloody stools and worsening anemia. CMV serologies were obtained, with CMV Immunoglobulin M and Immunoglobulin G positivity. Corticosteroids were discontinued given lack of response. She initiated a quadruple antibiotic regimen (amoxicillin, metronidazole, ciprofloxacin, and vancomycin) without clinical improvement. Anemia subsequently worsened requiring blood transfusions, and a third infliximab infusion was administered on induction Day 14 without clinical response within 5 days of administration, consistent with primary nonresponse to anti‐tumor necrosis factor‐α (TNF) therapy. Following agreement with our patient's family, our colorectal surgeons were consulted to further the discussion about a potential subtotal colectomy and ileostomy, while simultaneously a flexible sigmoidoscopy was planned to reevaluate disease and evaluate for CMV colitis.

**Figure 1:**
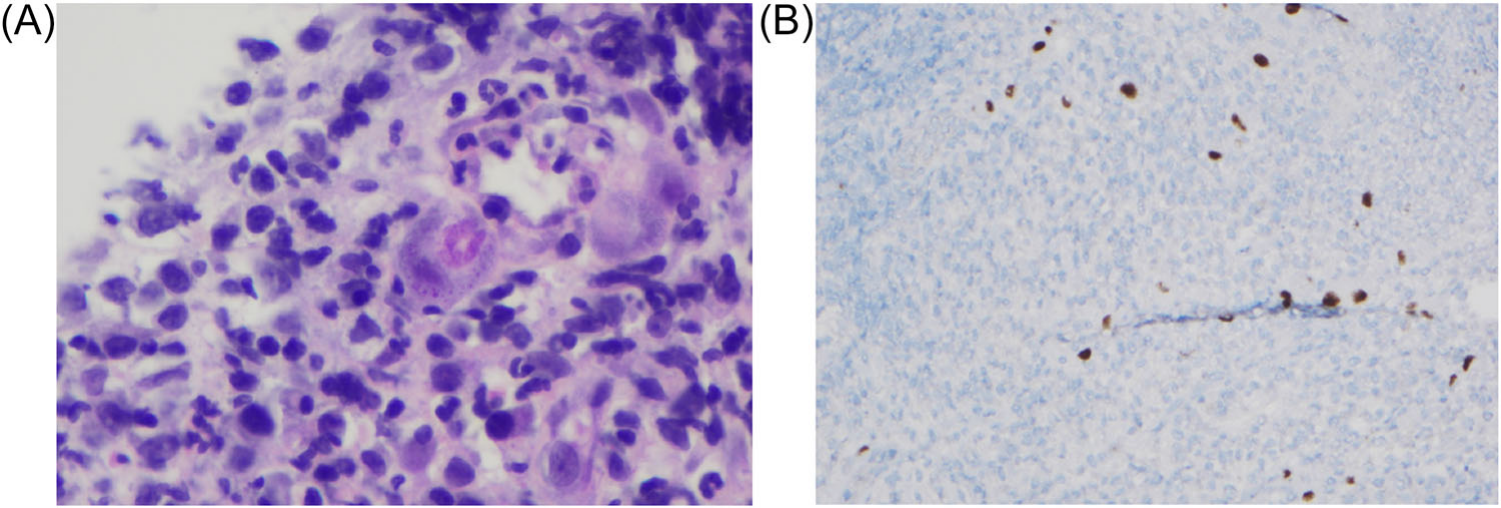
Histopathology from flexible sigmoidoscopy after corticosteroid and anti‐TNF failure. (A) H&E staining with an inclusion body. (B) Immunohistochemical stain positive for CMV. CMV, cytomegalovirus. H&E, hematoxylin and eosin; TNF, tumor necrosis factor.

**Figure 2:**
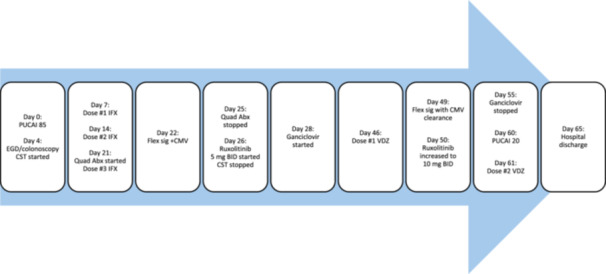
Clinical time course. Abx, antibiotics; CMV, cytomegalovirus; CST, corticosteroids; EGD, endoscopy; Flex sig, flexible sigmoidoscopy; IFX, infliximab; PUCAI, pediatric ulcerative colitis activity index; Quad, quadruple; VDZ, vedolizumab.

Flexible sigmoidoscopy redemonstrated severe colitis. Shared decision making with the family concluded that a third‐line medical therapy was strongly preferred to surgical intervention. Due to persistent severe disease activity, therapy was transitioned to ruxolitinib 5 mg twice daily. Histopathology of colonic biopsies identified severely active colitis with viral cytopathic effect on hematoxylin and eosin (H&E) staining—confirmed CMV with immunohistochemistry (Figure 1). Serum PCR found CMV viremia, and intravenous ganciclovir was started. Intermittent blood transfusions and parenteral nutrition were required while attempting to clear CMV. Despite a 3‐week course of antiviral treatment and confirmed clearance of CMV viremia, ASC symptoms persisted. Vedolizumab (VDZ) 10 mg/kg was administered for concomitant therapy without additional systemic immunosuppression, and 3 days later endoscopic reassessment was consistent with persistent severe colitis with clearance of CMV from colonic tissue. The ruxolitinib dose was increased to 10 mg twice daily. Within 2 weeks of starting vedolizumab and intensifying the ruxolitinib dose, significant clinical response was achieved allowing for hospital discharge (Figure 2). More than 12 months posthospitalization on this advanced combination therapy regimen, she has achieved endoscopic healing and histologic remission.

## 3 DISCUSSION

We report a case of a 4‐year‐old with newly diagnosed VEO‐IBD refractory to corticosteroids and infliximab. Her course was complicated by CMV colitis, and she was able to achieve clinical remission with a combination salvage therapy of ruxolitinib and vedolizumab. While it is unknown if the CMV infection was a primary infection or reactivation, immunosuppressive therapy is a known risk factor, and CMV colitis was identified after treatment with both steroids and infliximab. She continued on ruxolitinib while CMV was treated, which did not impair CMV clearance; the dose of ruxolitinib was intensified once CMV was cleared.

Inpatient management of ASC has a shifting treatment paradigm for therapy positioning. JAK inhibition, particularly tofacitinib and upadacitinib, is gaining interest as rescue therapy for hospitalized patients with ASC.^3^ Ruxolitinib is a selective JAK1 and JAK2 Janus Kinase inhibitor, currently approved to treat graft‐versus‐host disease in children 12 years of age and older and previously reported to induce remission in young children with VEO‐IBD.^4^ Ruxolitinib was chosen for its practical advantage in the young patient as a tablet that can be crushed as compared to upadacitinib, which must be swallowed whole. Liquid formulation of upadacitinib or tofacitinib was not available through our health system pharmacy or through insurance coverage. At this time, the choice of available JAK inhibitors may rely on circumstances of availability and tolerability to the patient, but the authors would consider all three of these JAK inhibitors as viable options. The adverse event profile of ruxolitinib mirrors the adverse risks of other JAK inhibitors used in inflammatory bowel disease practice, including infection, dyslipidemia, malignancy, cytopenias, increased aminotransferases, major adverse cardiovascular events, and thrombosis. While considering a colectomy, salvage therapy with a JAK inhibitor following inadequate response to anti‐TNF therapy is an emerging option in ASC. However, data continues to be sparse on the use of JAK inhibitors in very young children, and its positioning in the VEO‐IBD population remains undefined. In this case, the parents asked for medical therapy within the parameters of safety before proceeding to surgery. The treating team felt available data supported initiation of JAK inhibitor therapy in a young child who remained stable with the supporting therapy provided, however initiation of ruxolitinib should remain a case‐by‐case basis given the limited clinical data and novelty of this agent in treating VEO‐IBD. The patient did not experience any known complications or adverse events from ruxolitinib.

VDZ was initiated at the end of her antiviral course to concomitantly induce remission while minimizing further immunosuppression, given the lack of clinical improvement following CMV clearance. It is unknown if ruxolitinib would have induced the same remission as monotherapy, or if VDZ added a synergistic benefit working through a second immunologic pathway. Vedolizumab has a slow onset of action rendering its use limited in the ASC setting. However, some investigators have reported potential benefit in combination with another rapidly acting agent.^5^ We continued both medications in combination as initial maintenance therapy because of the refractory nature of her disease and excellent clinical response. More than 1 months posthospitalization, her fecal calprotectin reduced to 59 µg/g. While prophylaxis with valganciclovir was considered, it was not initiated. She continues to remain in remission without recurrence of symptoms. De‐escalation to a monotherapy regimen will be an anticipated goal if she can maintain a durable remission.

## 4 CONCLUSION

We report a case of a 4‐year‐old with newly diagnosed VEO‐IBD, who presented with ASC and achieved a colectomy free, clinical remission with ruxolitinib and vedolizumab combination therapy after nonresponse to corticosteroids and infliximab. While her course was complicated by CMV colitis, she was able to achieve clinical remission and clearance of her CMV infection while on this combination therapy.

## CONFLICT OF INTEREST STATEMENT

The authors declare no conflicts of interest.

## ETHICS STATEMENT

The parents of the child in question are aware of this report and have given their written informed consent.
